# Rapid identification of lactic acid bacteria at species/subspecies level via ensemble learning of Ramanomes

**DOI:** 10.3389/fmicb.2024.1361180

**Published:** 2024-04-08

**Authors:** Yan Ren, Yang Zheng, Xiaojing Wang, Shuang Qu, Lijun Sun, Chenyong Song, Jia Ding, Yuetong Ji, Guoze Wang, Pengfei Zhu, Likun Cheng

**Affiliations:** ^1^School of Life Science and Technology, Inner Mongolia University of Science and Technology, Baotou, China; ^2^Inner Mongolia Key Laboratory for Biomass-Energy Conversion, Baotou, China; ^3^Single-Cell Center, CAS Key Laboratory of Biofuels, Shandong Key Laboratory of Energy Genetics, Qingdao Institute of BioEnergy and Bioprocess Technology, Chinese Academy of Sciences, Qingdao, Shandong, China; ^4^Qingdao Single-Cell Biotechnology Co., Ltd., Qingdao, Shandong, China

**Keywords:** Ramanome, rapid classification, deep learning, LAB species/subspecies, fermented food

## Abstract

Rapid and accurate identification of lactic acid bacteria (LAB) species would greatly improve the screening rate for functional LAB. Although many conventional and molecular methods have proven efficient and reliable, LAB identification using these methods has generally been slow and tedious. Single-cell Raman spectroscopy (SCRS) provides the phenotypic profile of a single cell and can be performed by Raman spectroscopy (which directly detects vibrations of chemical bonds through inelastic scattering by a laser light) using an individual live cell. Recently, owing to its affordability, non-invasiveness, and label-free features, the Ramanome has emerged as a potential technique for fast bacterial detection. Here, we established a reference Ramanome database consisting of SCRS data from 1,650 cells from nine LAB species/subspecies and conducted further analysis using machine learning approaches, which have high efficiency and accuracy. We chose the ensemble meta-classifier (EMC), which is suitable for solving multi-classification problems, to perform in-depth mining and analysis of the Ramanome data. To optimize the accuracy and efficiency of the machine learning algorithm, we compared nine classifiers: LDA, SVM, RF, XGBoost, KNN, PLS-DA, CNN, LSTM, and EMC. EMC achieved the highest average prediction accuracy of 97.3% for recognizing LAB at the species/subspecies level. In summary, Ramanomes, with the integration of EMC, have promising potential for fast LAB species/subspecies identification in laboratories and may thus be further developed and sharpened for the direct identification and prediction of LAB species from fermented food.

## 1 Introduction

Lactic acid bacteria (LAB) are important members of the probiotic family and are often used as starter cultures of dairy products with important economic and nutritional value. “Identification of starter culture organism(s)” is a primary prerequisite for documenting the microbiological safety of LAB (Zhang et al., 2023). Several approaches have been developed to identify LAB strains. Classical phenotypic identification methods for LAB are based on a combination of their morphological and physiological characteristics (Jarocki et al., 2020). Genotype-based identification techniques include those based on 16S ribosomal RNA (16S rRNA) gene sequence, housekeeping genes (e.g., phenylalanyl-tRNA synthase alpha subunit, RNA polymerase alpha subunit, β-tubulin, and calmodulin), genome-wide single-nucleotide polymorphisms (SNPs), multilocus sequence typing (either core genome or whole-genome), or amplified fragment length polymorphism (Keith and Jolley, 2014; Mareike et al., 2014; Morovic et al., 2016; Lugli et al., 2019). Additionally, matrix-assisted laser desorption/ionization time-of-flight mass spectrometry (MALDI-TOF-MS), Fourier Transform Infrared (FT-IR) spectroscopy, and Tandem Mass Spectrometry (MS/MS) are frequently used for identification (Wenning et al., 2014). Although these methods have been well developed and proven to be reliable, they have several disadvantages. First, these methods usually require a culture-based strain isolation step that can take up to seven days (even longer for slow-growing cells), thus greatly delaying the time to report. Second, most of these methods are invasive and living cells must be broken down to extract DNA or proteins for identification. Third, these methods cannot be used to identify LAB in real-world settings. Accordingly, a fast, culture-independent, non-invasive and low-cost identification approach is highly desirable.

Single-cell Raman spectroscopy (SCRS) can provide the “molecular fingerprint” of a cell, and cells with different phylogenetic backgrounds can potentially be distinguished despite their varied metabolic states (He et al., 2019). SCRS is culture-independent and can promote “phenotype detection before culture.” In a previous study (Teng et al., 2016), we introduced the concept of Ramanome, the collection of SCRS data sampled from a cell population or consortium, as a type of single-cell-resolution metabolic phenome, and demonstrated the ability of SCRS to rapidly classify microbial species in a culture-free and label-free manner. Since its proposal in 2016, Ramanome has been applied in many microbial fields, including species classification (usually via pure cultures) and metabolic feature identification (Lu et al., 2020; Heidari et al., 2021; Liu et al., 2022). However, most previous studies have focused on microalgae or pathogens, but not LAB species or subspecies. Furthermore, Ramanomes are sensitive to not only “phylogeny” but also the “state” of a cell, yet existing experimental designs have generally failed to distinguish them. Consequently, a broadly applicable and reliable approach for the identification of LAB remains unexplored. The typical Ramanome of a single microbial cell contains thousands of variables based on wavenumbers. Thus, the greatest challenge in reliable microbial classification using Ramanome analysis is to retrieve characteristic information for each species that is normally not evident. Therefore, accurate species classification requires the application of advanced statistical algorithms to recognize differences in the Ramanomes of different species.

In recent years, researchers have begun to focus on the interpretability of machine learning models, which can significantly improve prediction accuracy and are more credible (Guo et al., 2021; Barton et al., 2022). To further improve the classification accuracy and stability, we adopted a model integration strategy to build an ensemble meta-classifier (EMC). The EMC is a blend of base classifiers (linear discriminant analysis (LDA), linear support vector machine (SVM), random forest (RF), extreme gradient boosting (XGBoost), k-nearest neighbors (KNN), partial least squares discriminant analysis (PLS-DA), convolutional neural network (CNN), and long short-term memory network (LSTM)) for each training performance compared with base classifiers, which has proven the application value of model integration in the Raman spectrum (Heidari et al., 2021). Although much research has employed Raman spectroscopy to identify bacteria, less research has been conducted on LAB. In this study, we established a method that combined Ramanome and EMC to discriminate the following nine closely related LAB species/subspecies: *Lacticaseibacillus paracasei* subsp. *paracasei*, *Lacticaseibacillus paracasei*, *Lacticaseibacillus rhamnosus*, *Lactiplantibacillus plantarum*, *Lactiplantibacillus plantarum* subsp. *plantarum*, *Lactiplantibacillus argentoratensis*, *Lactiplantibacillus pentosus*, *Lactobacillus gallinarum* and *Pediococcus pentosaceus*. We then compared the classification of nine machine-learning algorithms, and the results showed that the EMC model was the best classifier, with an average prediction accuracy of 97.3%, which was 3.66% higher than the maximum accuracy of the CNN of the single deep-learning model. This EMC utilizes the Ramanome approach for rapidly identifying single LAB cells, greatly accelerating the mining of LAB from fermented food.

## 2 Materials and methods

### 2.1 Chemical and biological materials

Nine LAB strains belonging to nine different LAB species/subspecies were collected from fermented dairy samples (Table 1). A total of 1,650 Raman spectrum fingerprints (600–1800 cm^−1^) were obtained for all of the bacterial species/subspecies. All LAB strains were stored in the School of Life Science and Technology, Inner Mongolia University of Science and Technology, Baotou, China, after isolation. These strains were identified using biochemical methods and 16S rRNA and stored in a ThermoFisher freezer at −80°C.

**Table 1:** LAB samples.

No.	Strain number	LAB species/subspecies
001	IMUST00001	*Lacticaseibacillus paracasei subsp. paracasei*
016	IMUST00016	*Lacticaseibacillus paracasei*
036	IMUST00036	*Lactobacillus gallinarum*
063	IMUST00063	*Lactiplantibacillus plantarum*
067	IMUST00067	*Lactiplantibacillus plantarum subsp. plantarum*
114	IMUST00114	*Lactiplantibacillus argentoratensis*
138	IMUST00138	*Pediococcus pentosaceus*
143	IMUST00143	*Lacticaseibacillus rhamnosus*
146	IMUST00146	*Lactiplantibacillus pentosus*

### 2.2 SCRS acquisition and parameter setting

After culturing each strain on MRS agar plates overnight, single colonies were inoculated in 5 mL MRS broth. Then, 1% bacterial culture was inoculated into a new MRS broth culture and incubated at 37°C for 16 h at 200 rpm. Thereafter, 1 mL sample from each strain was centrifuged at 8000 rpm for 2 min and washed thrice with sterile water. Sterile water samples with a moderate-weighted drop hanging on a calcium fluoride (CaF_2_) slide were air-dried prior to Raman analysis.

All SCRS data were acquired using a RACS instrument (Qingdao Single-Cell Biotechnology, Qingdao, China). The system is equipped with a microscope with a 100 × dry objective (NA = 0.80) and a 532 nm Nd: YAG laser with a maximum power of 100 mW. Each cell was exposed to a laser for 1 s, and the spectra were recorded using a diffraction grating with 300 grooves/mm. A total of 180 cells were analyzed using SCRS for each biological replicate of each LAB strain.

### 2.3 Data preprocessing and machine learning

To process and analyze the Ramanome data effectively, we adopted the following comprehensive processing flow to ensure consistency, comparability, and reliability of the data (Guo et al., 2021). Our processing pipeline primarily included baseline correction, spectral smoothening, and normalization, which played key roles in extracting spectral features and reducing noise (Senger and Scherr, 2020). Firstly, we use the polynomial fitting method to perform baseline correction on the spectrum. By estimating the background baseline of the spectrum and subtracting it from the original spectrum, we could reduce the background interference and highlight the characteristics of the spectral peak. Then, to further eliminate the noise in the data, we applied the Savitzky–Golay smoothing method to smooth the spectral data by fitting polynomials, so as to retain important information while reducing unnecessary fluctuations. In the final stage of spectrum pre-processing, we employed subsequent analysis, a step that involves dividing each data point of the spectral data by the maximum value of that spectrum, mapping the data to between 0 and 1, thus achieving a uniform amplitude scale. The pre-processing diagram is shown in Supplementary Figure S1. After preprocessing the SCRS data, the average SCRS data for each bacterial species/subspecies were generated by calculating the intensities at each Raman shift, and standard deviations (shaded error bands) were also calculated. The averaged SCRS data were imported into the LabSpec software (HORIBA Scientific, Japan).

### 2.4 Comparative evaluation of machine learning algorithms

A comprehensive list of machine-learning techniques, including six machine-learning models (LDA, SVM, RF, XGBoost, KNN, and PLS-DA) and two deep-learning models (CNN and LSTM), was employed to generate the base machine-learning classifiers. Metrics (Accuracy, Mean Sensitivity, Mean Specificity, Kappa) are essential for evaluating the effects of machine-learning algorithms during data analysis. In this study, different evaluation metrics were used to measure the performance indicators in spectrum-signal recognition.

## 3 Results

### 3.1 Average SCRS spectra and characteristic peaks

**Figure 1:**
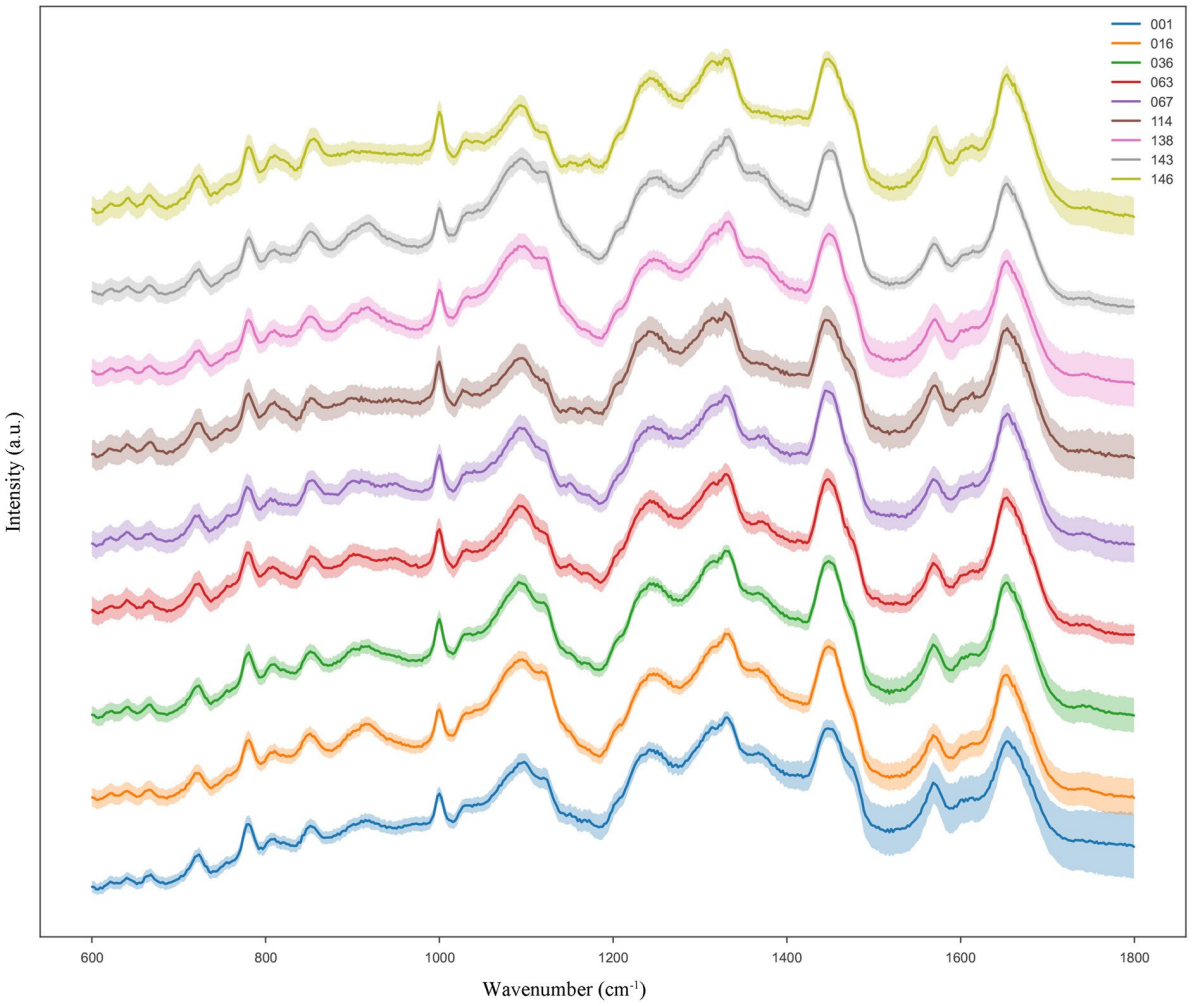
Typical Ramanome of the nine LAB species/subspecies. The mean Raman spectra are indicated by the solid line (colored), and the standard deviations are represented by the shadow. For each species/subspecies, the Ramanomes of more than 160 single cells were acquired via SCRS analysis.

In this study, we calculated the average Raman intensity at each Raman shift to generate the average SCRS data for each of the nine LAB species/subspecies. Hence, we built a reference Ramanome database that spans a wide range of nine LAB species/subspecies. Notably, nine LAB species/subspecies from each of the genera *Lacticaseibacillus*, *Lactiplantibacillus*, *Lactobacillus*, and *Pediococcus* were included to determine the feasibility of the species/subspecies-level classification (Table 1). Each species was cultured in triplicates under optimal growth conditions. From each biological replicate culture, 60 cells were randomly selected for SCRS spectrum acquisition as Ramanomes; thus, 180 cells were sampled per species. Different LAB species show differences in their Raman intensities and distributions of their characteristic peaks, which can be used to discriminate them. In addition, the standard error band was visualized in the averaged Raman spectrum of each bacterium to determine whether the spectral data exhibited good repeatability during SCRS spectrum generation. The narrower the error band, the higher the repeatability of the Raman spectrum. According to the results shown in Figure 1, the reproducibility of the Raman spectra for each LAB species/subspecies was good.

**Figure 2:**
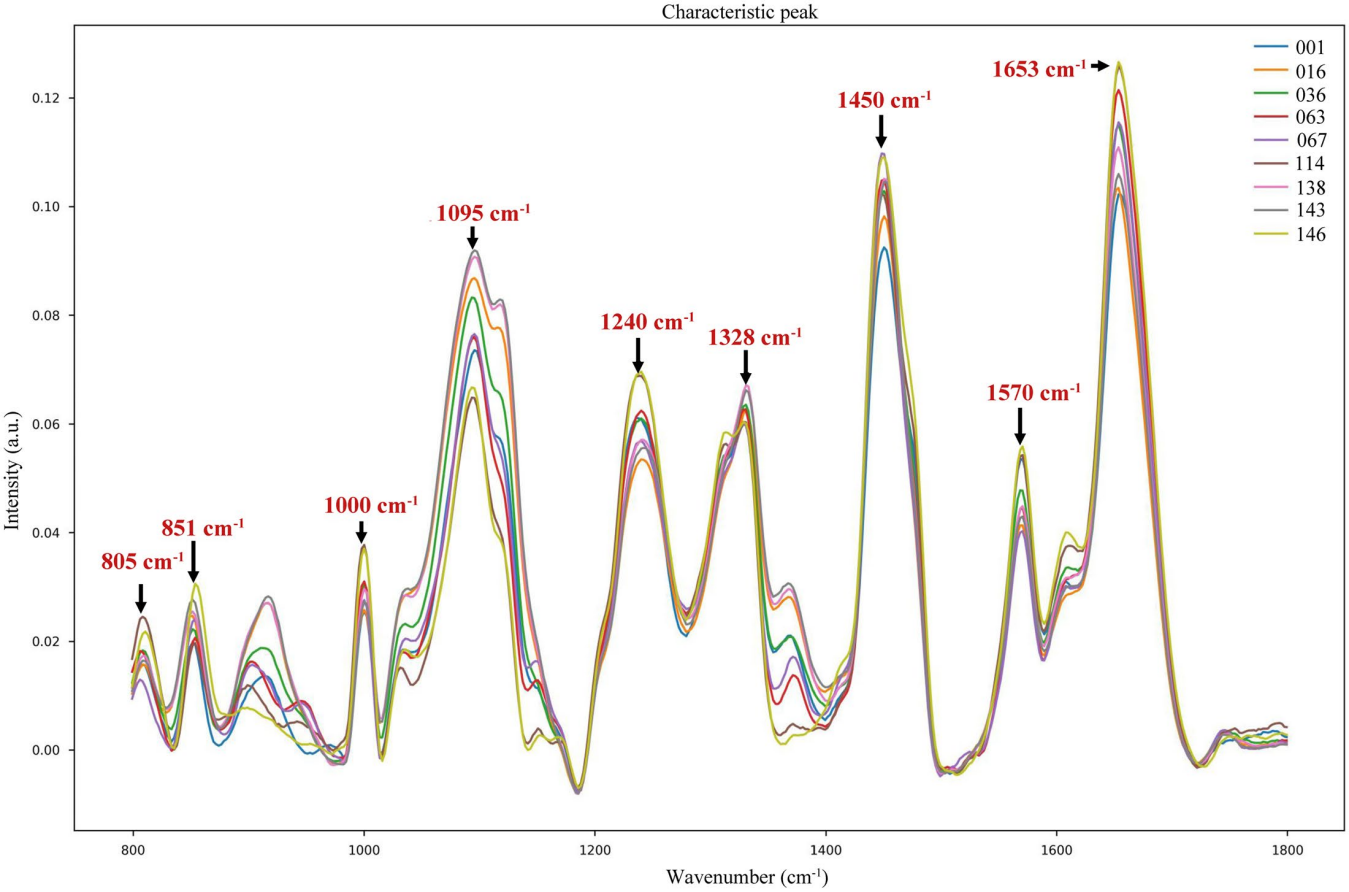
Characteristic peaks of different LAB species/subspecies in the fingerprint region.

We found that the spectra of the nine samples were different at the wave intensities of 805, 851, 1,000, 1,095, 1,240, 1,328, 1,450, 1,570, and 1,653 cm^−1^ (Figure 2). The specific characteristic peaks and their corresponding biological meanings are listed in detail in Table 2. Distributions of the characteristic peaks of all nine LAB species and subspecies are shown in Supplementary Figure S2A. The difference in the significance of each characteristic peak is shown in Supplementary Figure S2B, where the value represents the degree of significance (0, *p* > 0.05; 1, 0.01 < *p* ≤ 0.05; 2, 0.001 < *p* ≤ 0.01; 3, 0.0001 < *p* ≤ 0.001; 4, *p* ≤ 0.0001). All bacteria share basic structures, such as cell walls and cell membranes, but the composition and types of proteins, lipids, and nucleic acids vary depending on the species or subspecies (Rodriguez et al., 2017). Proteins make up 40–50% of bacterial cells. The characteristic Raman peaks at 851 and 1,000 cm^−1^ were associated with proline, tyrosine, and phenylalanine. The amide I band of the proteins (1,653 cm^−1^) contributed substantially to the accurate discrimination of the nine LAB species/subspecies. Lipids make up 10–15% of bacterial cells. The lipid of the CH_2_ bending was associated with 1,450 cm^−1^. Bacterial cells contain 2–4% DNA and 5–15% RNA. The PO_2_^−^ symmetric stretching and PO_2_^−^ asymmetric phosphate were associated with 1,095 cm^−1^ and 1,240 cm^−1^, while the characteristic Raman peaks at 805 and 1,328 cm^−1^ were due to backbone geometry, phosphate ion interactions, and CH_3_CH_2_. Furthermore, the Raman peak at 1570 cm^−1^ was attributed to guanine or adenine (Wang et al., 2016; Sun et al., 2023).

**Table 2:** Biological meaning of the characteristic peaks for the nine LAB species as per the literature.

Peak (cm^−1^)	Assignment	References
805	Backbone geometry and phosphate ion interactions	Chan et al. (2006)
851	Proline, hydroxyproline, and tyrosine	Laska and Widlarz (2005)
1,000	Phenylalanine	Wang et al. (2016) and Stone et al. (2004)
1,095	Lipid	Loan et al. (2004)
1,240	Asymmetric phosphate [PO^2−^(asym.)] stretching modes	Chan et al. (2006)
1,328	CH_3_CH_2_ wagging mode in purine bases of nucleic acids	Wang et al. (2016)
1,450	CH_2_ bending	Jyothi Lakshmi et al. (2002)
1,570	Guanine, adenine, and TRP (protein)	Stone et al. (2004)
1,653	amide I	Farquharson et al. (2005)

**Figure 3:**
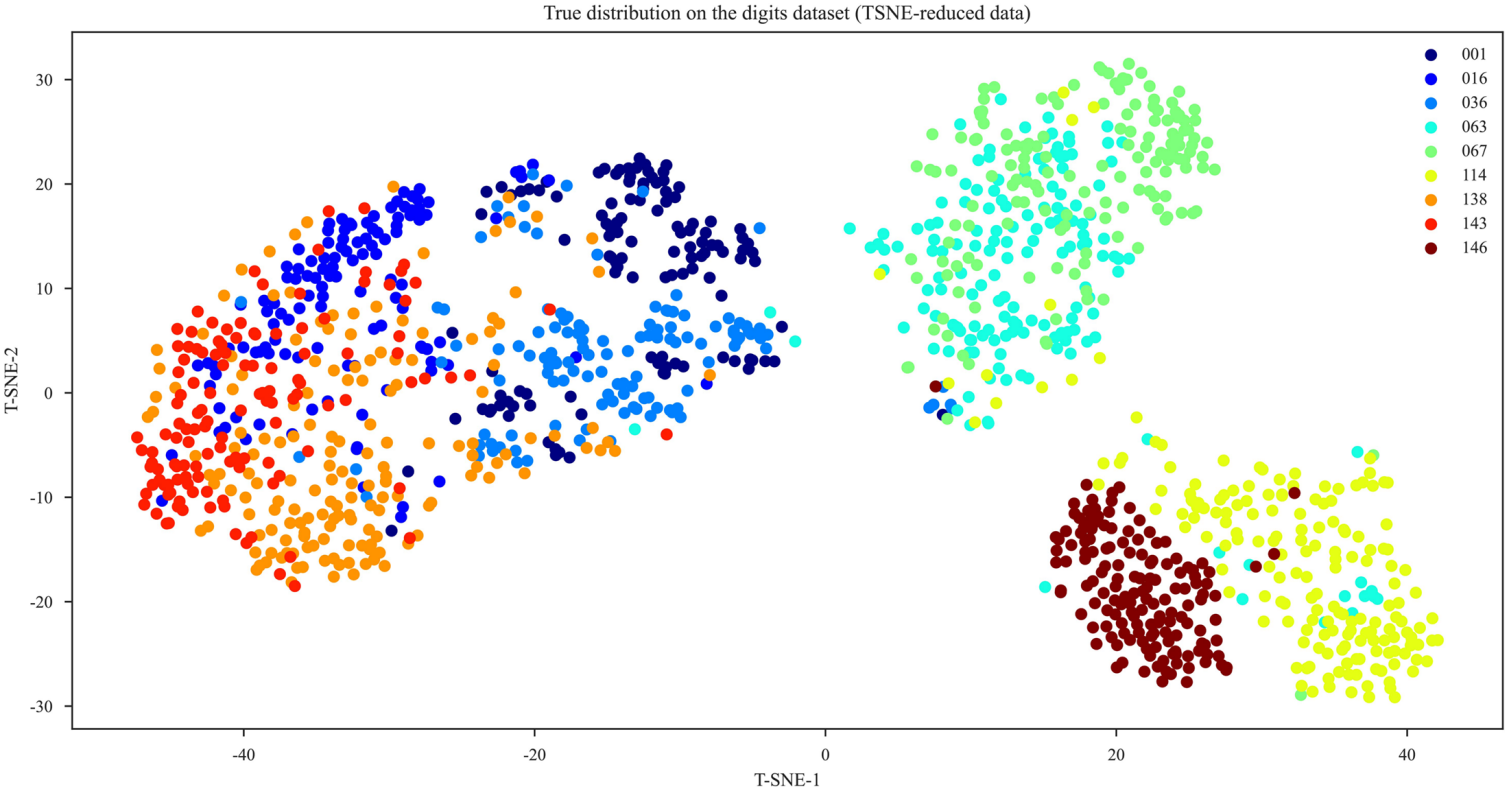
T-SNE cluster analysis of the nine LAB species/subspecies.

We used t-distributed Stochastic Neighbor Embedding (t-SNE) to cluster the nine species/subspecies (Figure 3). T-SNE cluster analysis results showed that strains 001 (*Lacticaseibacillus paracasei* subsp. *paracasei*), 036 (*Lactobacillus gallinarum*), and 016 (*Lacticaseibacillus paracasei*), (*Pediococcus pentosaceus*) and 143 (*Lacticaseibacillus rhamnosus*) had many overlapping regions that could not be distinguished but could be distinguished from other strains; strains 063 (*Lactiplantibacillus plantarum*) and 067 (*Lactiplantibacillus plantarum* subsp. *plantarum*) could not be distinguished but could be distinguished from the other strains; and strains 114 (*Lacticaseibacillus argentoratensis*) and 146 (*Lacticaseibacillus pentosus*) could be distinguished from the other strains. Therefore, accurate differentiation was not possible when comparing the Raman spectra of the nine LAB species/subspecies. To overcome this difficulty, six machine-learning and two deep-learning models were built to identify the nine LAB species/subspecies.

### 3.2 Machine learning analysis and ensemble meta-classifier

**Figure 4:**
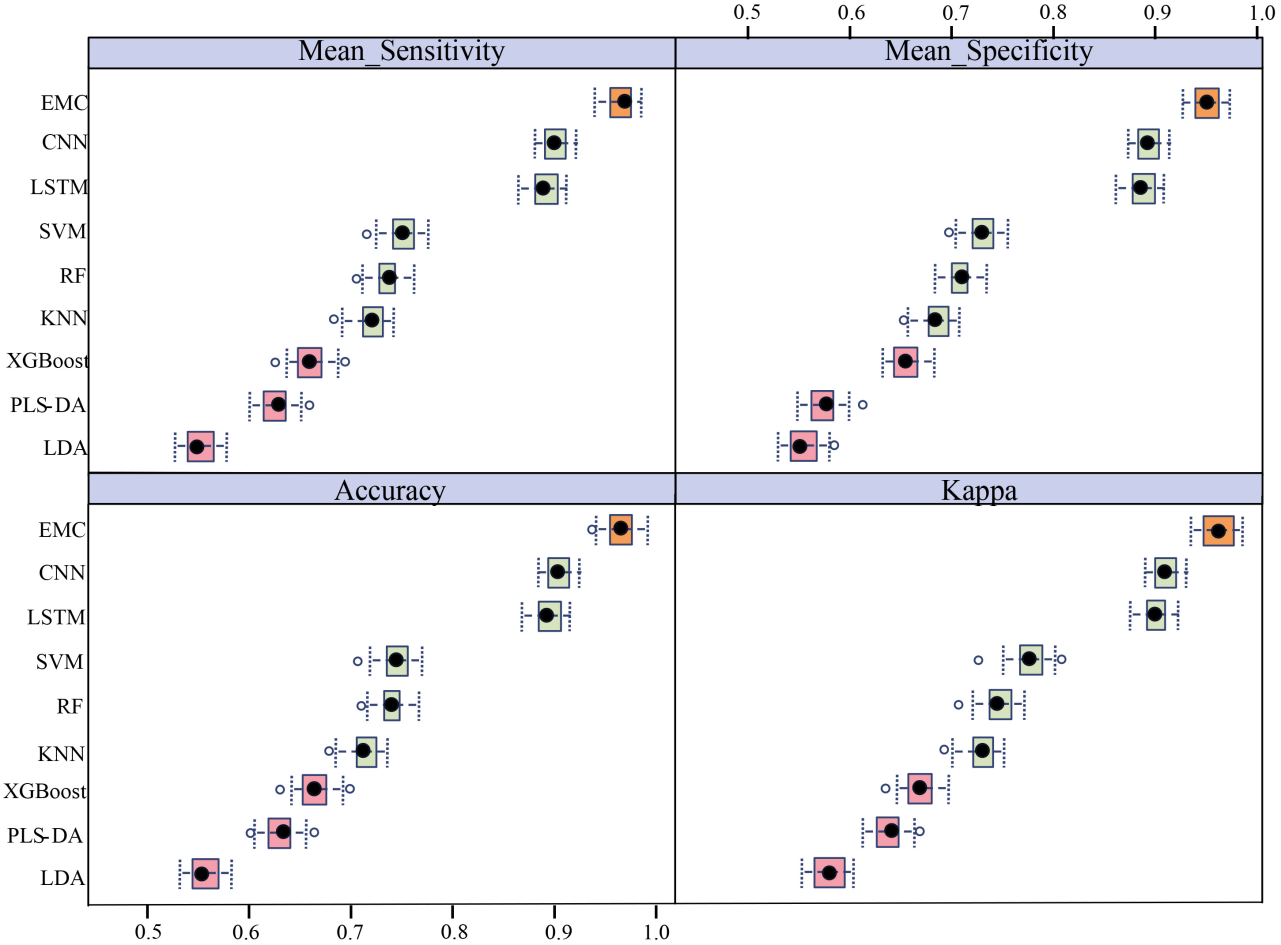
Phylogenetic classification of LAB based on machine learning. Results are based on 10 times 10-fold cross-validation. The box plots illustrate the distribution of the 100 resamples, with the central dot showing the median and the whiskers representing the quartiles. The orange box plot represents the EMC model, green box plots represent the first five classifiers with the best performances that were included in the EMC model, and pink box plots represent the three classifiers that performed poorly.

In this study, six machine learning models (LDA, PLS-DA, XGBoost, KNN, RF, and SVM) and two deep learning models (LSTM and CNN), with eight independent classification models, were employed for the classification of LAB Ramanome data. Each model exhibited a specific classification performance during separate training and validation (70% for the training set and 30% for the test set). Four evaluation metrics, accuracy (ACC), mean sensitivity, mean specificity, and kappa, were applied to measure the performance of all machine learning models (Figure 4). Ten-fold cross-validation was used to determine whether the models were overfitted during training. The results are shown in Table 3. The accuracy of the eight classifiers was between 70.41 and 93.64%. LSTM and CNN achieved comparatively good classification results with accuracies of 92.07 and 93.64%, respectively. Meanwhile, the specificity of the eight classifiers was between 69.07 and 93.14%. LSTM and CNN also achieved comparatively good classification results with specificities of 91.77 and 93.14%, respectively. A schematic illustration of the CNN and LSTM analyses of the Ramanome data is shown in Supplementary Figures S3A,B. Excluding the LDA, PLS-DA, and XGBoost algorithms, the KNN and RF achieved an accuracy and a specificity of more than 80%.

**Table 3:** Comparison of the predictive abilities of the nine supervised learning algorithms at LAB species/subspecies level.

Metrics	LDA	PLS-DA	XGBoost	KNN	RF	SVM	LSTM	CNN	EMC
Accuracy (%)	70.41	73.11	76.59	80.76	82.45	84.32	92.07	93.64	97.3
Mean sensitivity (%)	70.78	74.85	76.33	81.23	81.94	83.97	91.89	93.32	97.54
Mean specificity (%)	69.07	73.37	75.46	80.31	81.07	83.03	91.77	93.14	96.96
Kappa	0.6871	0.7194	0.7532	0.8066	0.8184	0.8437	0.9265	0.9416	0.9836

The sensitivity and specificity of each model were similar to the accuracy results. To further validate the performance of different machine learning models for different bacterial species/subspecies, we used kappa values to measure the specificity and sensitivity of each model. The larger the kappa value, the better the performance of the model. By comparing the accuracy (93.64%), sensitivity (93.32%), specificity (93.14%), and kappa (0.9416), we concluded that the CNN model had the best performance for Raman data analysis at the bacterial species/subspecies level. The model with a similar predictive performance was LSTM. The first five models with the best performances were CNN, LSTM, SVM, RF, and KNN. Conversely, the LDA, PLS-DA, and XGBoost models failed to correctly distinguish among the nine LAB species/subspecies.

**Figure 5:**
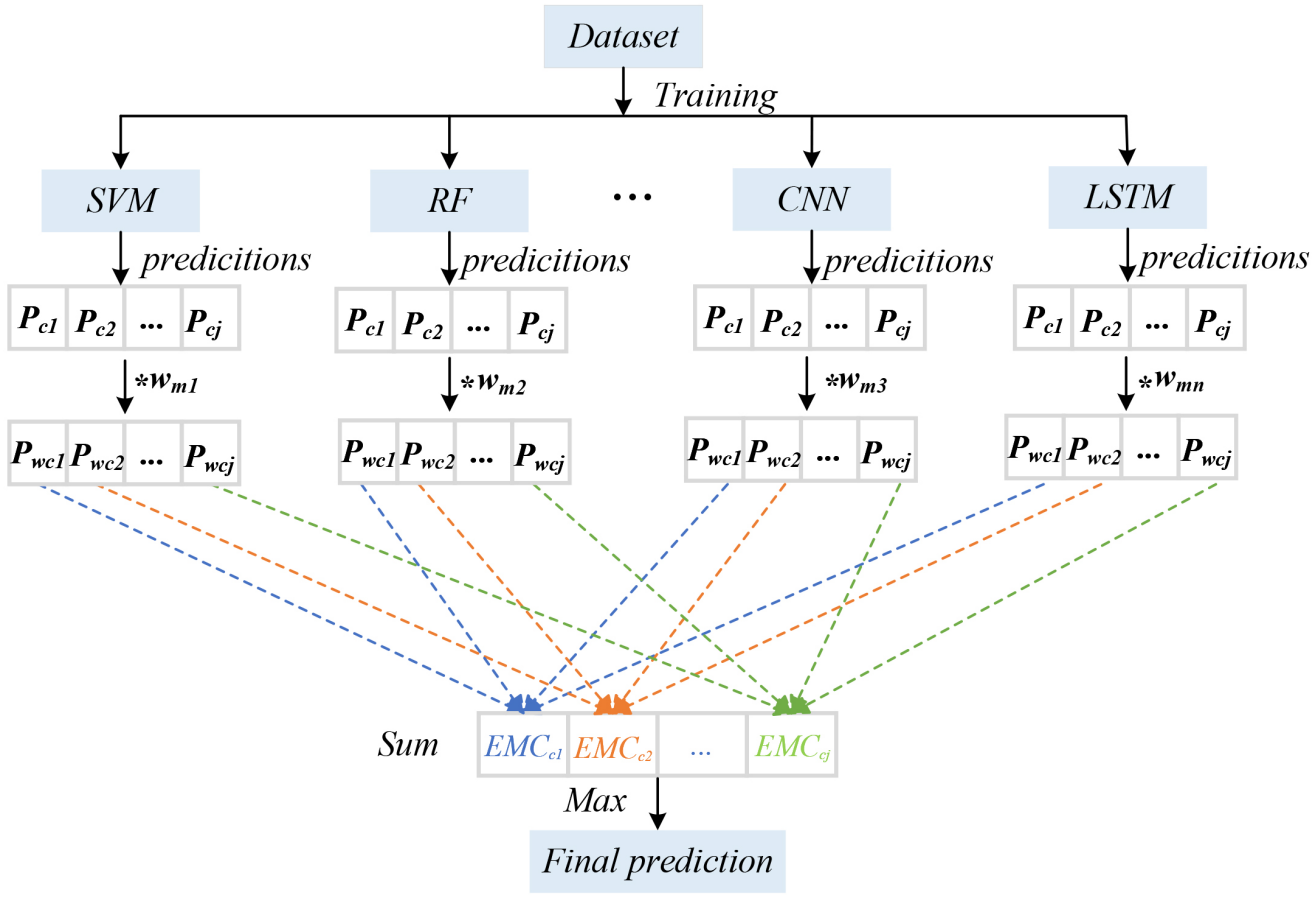
The diagram of EMC. We evaluated the performance of each classification model and used the accuracy of a single classifier for the dataset as a measurement index; took the accuracy rate as the weight of the model and normalized it; calculated the class probability of each classifier and carried out weighted summation; and considered the category with the greatest probability as the final prediction result. W*_mn_* represents the weight of the nth classification model; P*_cj_* represents the estimated probability of class *j*.

To further improve the accuracy and stability of the classification, we adopted the EMC strategy (Table 3). First, we evaluated eight models on the test set and their accuracies in the classification task. Each algorithm exhibited a certain classification performance during individual training and verification. By comparing the performance of the models, we chose the highest accuracy of the first five types of models (CNN, LSTM, SVM, RF, and KNN) as the components of the integration. During the integration process, we used a voting strategy to combine the predictions of various models (Figure 5). First, we (1) evaluated the performance of each classification model and used the accuracy of a single classifier of the dataset as a measurement index; (2) took the accuracy rate as the weight of the model and normalized it, where the specific calculation formula was as follows:

$$W_{mn}=\frac{\mathrm{Accurac}y_{mn}}{\mathrm{Sum}\left(\mathrm{Accurac}y_{m1},\mathrm{Accurac}y_{m2},\ldots ,\mathrm{Accurac}y_{mn}\right)}$$

with $W_{mn}$ representing the weight of the nth classification models (*m*), $\mathrm{Accurac}y_{mn}$ representing the accuracy rate of the nth classification model, and *Sum* representing the sum; (3) calculated the class probability of each classifier and carried out weighted summation, with $P_{cj}$ representing the estimated probability of class *j*. Each branch model calculates the confidence (prediction probability) for each category and considers the category with the highest confidence as the final prediction category; (4) considered the category with the greatest probability as the final prediction result. This method of model integration helps reduce the prediction bias of individual models, improves the stability and accuracy of the overall classification, and provides strong support for classification tasks in practical applications. This choice was based on the classification of different models into different categories based on comprehensive considerations. Next, we used the ensemble learning method to fuse the predictions of the five models and obtain a stronger classification performance. In the ensemble process, a voting strategy was adopted to combine the prediction results of each model. This method improved the stability and accuracy of the overall classification. The average accuracy of EMC was 97.3%, which was 3.66% higher than the highest accuracy of the single-model CNN (93.64%). The sensitivity, specificity, and kappa coefficient increased to 97.54, 96.96%, and 0.9836, respectively (Table 3). Hence, the EMC of the best-performing base classifiers was built, which performed better than each base classifier.

### 3.3 Confusion matrix analysis

**Figure 6:**
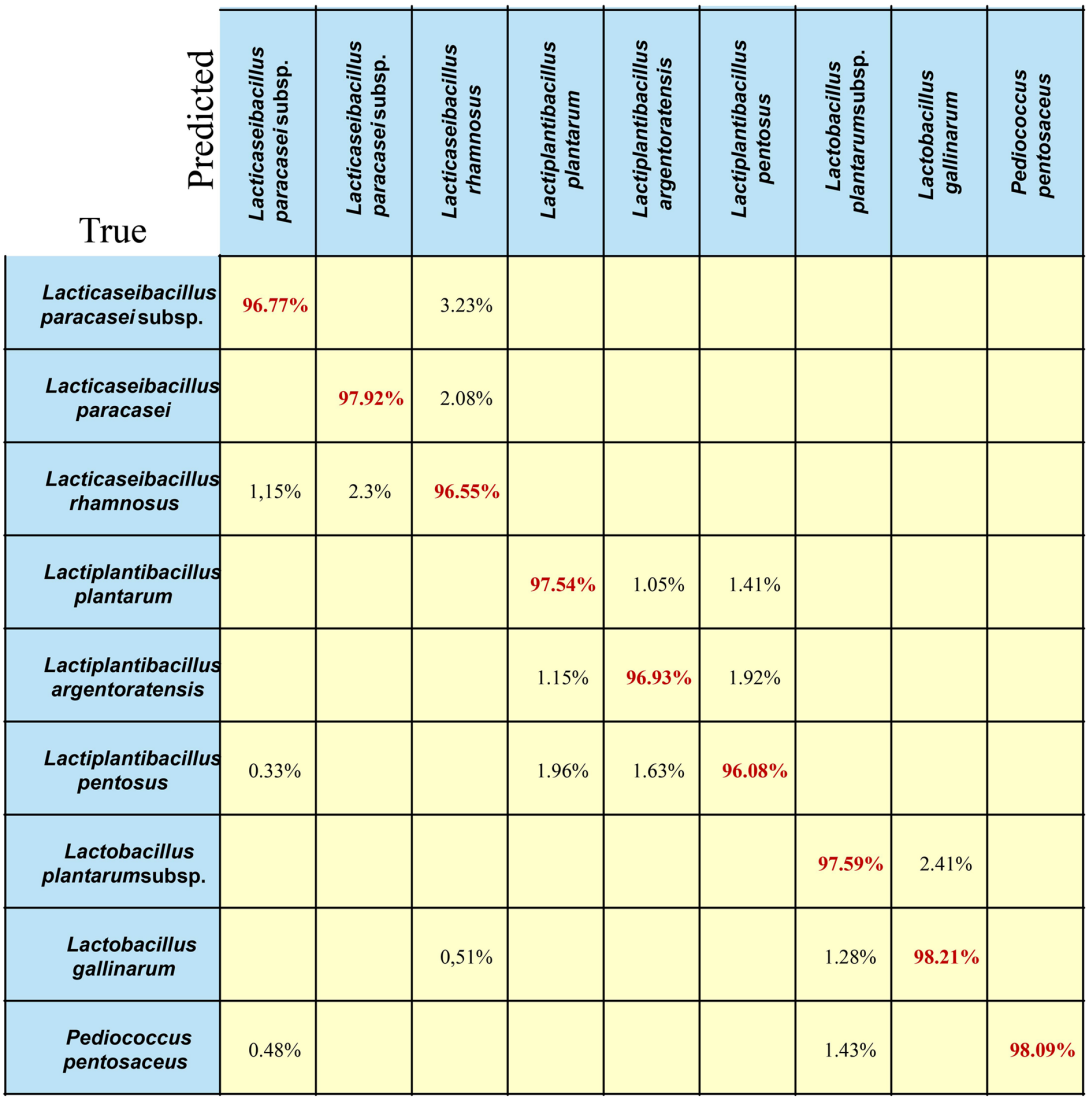
Confusion matrix of the EMC model for LAB species. Each row in the matrix represents an instance in the true class, and each column in the matrix represents an instance in the predicted class. The diagonal line represents the prediction accuracy of the EMC model on different bacterial strains. The average prediction accuracy of the EMC model is 97.3%.

A confusion matrix is a quantitative visualization method used in machine-learning analysis to summarize the prediction results of a classification model (Liang et al., 2022). A confusion matrix describes the relationship between the real attributes of the sample data and the predicted results. Therefore, it is an efficient method for evaluating the performance of machine learning classifiers. In this study, we chose the best-performing model (EMC) to calculate the confusion matrix for LAB species/subspecies. The results of cross-validation may be presented in the form of a confusion matrix, where the true class (rows of the matrix) corresponds to the identification of species/subspecies based on 16S rRNA and biochemical methods, and the predicted class (columns) corresponds to the identification suggested by the Ramanome (Figure 6). The correctly identified spectra (in red) are diagonal. Off-diagonal spectra (in black font) correspond to incorrectly identified spectra (and their suggested classification by the Ramanome). The sensitivity of the method (true-positive rate (True)) and false-negative rate are presented in the rightmost columns, which represent the relative counts of SCRS data of the given species/subspecies that were incorrectly identified. The two bottom rows represent the positive predictive values (Predict), the relative counts of correctly identified spectra and spectra from different species falsely identified as a given species in each column, and the false discovery rate. In an ideal case, the prediction should be 100%. The results for the testing set at the single-spectrum level are shown in Figure 6 (Katarina et al., 2023).

As shown in Figure 6, for the nine different LAB species/subspecies, the EMC model achieved good prediction ability, and the classification accuracy for each strain was greater than or equal to 97%. As an example, 96.55% of *Lacticaseibacillus rhamnosus* were correctly predicted, whereas 2.3% of *Lacticaseibacillus rhamnosus* were misclassified as *Lacticaseibacillus* paracasei, and 1.15% of *Lacticaseibacillus rhamnosus* were misclassified as *Lacticaseibacillus paracasei* subsp. *paracasei*. In addition, 1.15% of *Lactiplantibacillus plantarum* and 1.92% of *Lactiplantibacillus pentosus* were predicted to be *Lactiplantibacillus argentoratensis.* Notably, 1.43% of *Lactiplantibacillus plantarum* subsp. *plantarum* and 0.48% of *Lacticaseibacillus paracasei* subsp. *paracasei* was predicted to be *Pediococcus pentosaceus*.

## 4 Discussion

The core characteristics of LAB products depend on the state of live bacteria, quantity, and bacterial health function, and their function and safety are strain-specific. Therefore, the precise identification of LAB at the strain level has become increasingly important domestically and internationally. The species/subspecies of LAB can be identified by means of colony morphology, physiological and biochemical characteristics, molecular biological analysis, and MALDI-TOF-MS. Although the Ramanome has the potential for the rapid detection of bacterial pathogens and microalgae, little work has been done on LAB in both species and subspecies identification (He et al., 2019; Heidari et al., 2021). In addition, owing to the sophistication of Ramanome data, classical statistical methods are insufficient for spectral data analysis. Thus, Ramanome data with further EMC analysis is necessitated. The EMC in this study exhibited improved training performance metrics; the accuracy and sensitivity increased to 97%, while specificity and Kappa increased to 97 and 98%, respectively. Hence, the EMC of the best-performing base classifiers was built, which performed better than each base classifier. As expected, ensemble learning augmented the training performance for the classification of the nine LAB species, in which both accuracy and mean sensitivity increased to 97.3% and mean specificity increased to 97.54%.

Many previous studies have explored the possibility of combining the Ramanome technique with machine learning algorithms for the rapid detection of bacteria (Rodriguez et al., 2017; Lu et al., 2020; Liang et al., 2022; Rebrosova et al., 2022; Zhang et al., 2023). For example, Zhang et al. built a reference database of Ramanome from 21 pure-cultured probiotic strains that represent the standard statutory strains for human consumption (including 14 *Lactobacillus* spp., 6 *Bifidobacterium* spp., and 1 *Streptococcus* sp.), finding that the CNN classification algorithm showed that the best classification accuracy (93.02 ± 1.39%), indicating that Ramanome combined with machine learning is capable of discriminating probiotic bacteria (Zhang et al., 2023). However, little work on ensemble learning algorithms has been employed for SCRS analysis until recently, where Baladehi et al. combined EMC learning and Ramanome to rapidly identify and metabolically profile microalgal single cells and found that the accuracy of classifying species and states can be above 97%, further highlighting the promising potential of the technique for classification and identification of microorganisms (Heidari et al., 2021). It is interesting to note that in recent studies, the deep learning algorithm LSTM did not perform well as did CNN, and in some cases, it was not as good as classical algorithms, such as RF and KNN (Yu et al., 2021; Tang et al., 2022). However, in our study, the LSTM method was almost as accurate as the CNN method, with an accuracy of 92.07%. A recent study by Yu et al. (2021) showed that the LSTM model was faster and more accurate than the CNN model, achieving an average isolation level accuracy of more than 94%, which is worthy of further exploration (Yu et al., 2021).

In this study, we aimed to reveal the intrinsic differences between the Ramanomes of nine LAB species/subspecies using a novel EMC learning model. In addition, the prediction abilities of nine machine learning algorithms (one ensemble learning algorithm, EMC; two deep learning algorithms, CNN and LSTM; and six classical machine learning algorithms, LDA, PLS-DA, XGBoost, KNN, RF, and SVM) were thoroughly compared. A comparison of the evaluation indicators (accuracy, sensitivity, specificity, and Kappa) for all the machine learning algorithms (Table 3) clearly showed that the performance of the EMC algorithm was the best (97.3%), which improved by 3–4% compared with the other two deep learning models, CNN and LSTM. The results indicate that when data complexity increases, the novel EMC learning algorithm displays better robustness than classical machine learning algorithms, which is consistent with a previous report (Heidari et al., 2021). Therefore, the EMC learning algorithm combined with the Ramanome can classify and predict LAB at the species/subspecies level with high accuracy and computational efficiency.

Altogether, this novel approach could significantly accelerate the identification of LAB species/subspecies, leading to the timely and accurate treatment of similar LAB species. However, this study had some limitations. Only nine LAB species/subspecies were included. This small number might affect the robustness of the model and cause data overfitting. Based on this pilot test, we plan to perform a more extensive study to include more LAB strains/species, as well as more microbial species, in the testing of the method. For example, in our next experiment, we mocked the microbiota by combining *Escherichia coli* and a mixture stock containing equal amounts of the nine LAB strains in different ratios (1:99, 10:90, 50:50, 95:5, 99:1). For each of the five mocking LAB samples, 10 randomized SCRS-based identifying experiments were performed using the EMC model. Then, the proportion of *Escherichia coli* in each reconstructed simulated community was observed. Finally, the reliability of the model to distinguish *Escherichia coli* from other LAB strains was evaluated. These initial results can be improved by adding more samples to the database to increase the robustness of the model, which is especially important for the EMC to avoid overfitting the data. Expanding the number of species and subspecies in the database would allow us to further evaluate the capacity of the Ramanome to identify LAB at the species/subspecies level or even the strain level. Other possibilities to improve classification rates include selecting key wavelengths associated with differences in species and subspecies and building multilayered models to assess the species and subspecies. Developing a panel for inclusivity and exclusivity studies would help estimate the sensitivity and specificity of the method. Parallel analysis of panel strains with a gold-standard diagnostic tool would also help assess the potential of this method as an alternative to conventional techniques (Treguier et al., 2019).

Despite the limitations mentioned above, the Ramanome has numerous advantages, proving its high potential as an *in situ* diagnostic tool. It is non-invasive and non-destructive; therefore, samples (cells) can be used for further or complementary testing. It has a broad spectrum of applications across various scientific fields for numerous *in situ* diagnostics purposes. Moreover, the method does not require expensive consumables. Furthermore, the sample preparation is easy and quick. Therefore, we believe that the Ramanome can become an assistive tool in the future, significantly improving the accuracy of LAB identification.

## 5 Conclusion

This study presents a highly effective approach for the identification of LAB species/subspecies using Ramanome combined with EMC at the single-cell level. We demonstrated the ability of our method to distinguish closely related LAB species and subspecies with a high degree of accuracy. Moreover, this is the first time that the EMC algorithm has been used to analyze LAB. This tool may be useful for further investigations into the identification of different microorganisms. Hopefully, the miniaturization and automation of the Raman instrument, accumulation of more Ramanome data for different LAB species/subspecies, and fast cloud data services will promote the utilization of this technique in various applications for accurate LAB identification.

## Data availability statement

The raw data supporting the conclusions of this article will be made available by the authors, without undue reservation.

## Author contributions

YR: Writing – original draft, Writing – review & editing. YZ: Investigation, Methodology, Writing – review & editing. XW: Investigation, Methodology, Writing – review & editing. SQ: Investigation, Methodology, Writing – review & editing. LS: Data curation, Methodology, Visualization, Writing – review & editing. CS: Data curation, Methodology, Visualization, Writing – review & editing. JD: Data curation, Methodology, Visualization, Writing – review & editing. YJ: Data curation, Methodology, Visualization, Writing – review & editing. GW: Data curation, Methodology, Visualization, Writing – review & editing. PZ: Conceptualization, Project administration, Supervision, Writing – review & editing. LC: Conceptualization, Project administration, Supervision, Writing – review & editing.
