# Supplementary material for: Rapid identification of lactic acid bacteria at species/subspecies level via ensemble learning of Ramanomes
Source: Front Microbiol. 2024 Apr 8;15:1361180. doi: 10.3389/fmicb.2024.1361180 (PMC11033474; doi:10.3389/fmicb.2024.1361180)
Supplement: Supplementary file 1 [file Data_Sheet_1.zip › Supplemental Information.DOCX]

We have provided the parameters of six machine learning models in the Supporting Material for readers to understand and reference.

(1) Support Vector Machine (SVM): SVM is a supervised learning algorithm commonly used for classification and regression problems. In Raman spectral classification, SVM separates different classes of samples by finding an optimal hyperplane. It performs well on high-dimensional data, such as Raman spectral data, and is able to handle nonlinear relationships and achieve complex mappings through kernel functions. The advantages of SVM include excellent performance in small samples and high generalization ability. In this paper, the parameters of SVM were set as follows:

C parameter (penalty coefficient):1;

Kernel type: linear.

(2) Random Forest (RF): RF is an ensemble learning algorithm that classifies by building multiple decision trees. In Raman spectral classification, the RF reaches the final classification result by voting on the robustness of certain predications when dealing with noisy data. In this paper, RF parameters were set as follows:

Number of trees (n_estimators): 100;

Maximum tree depth (max_depth):20;

Column sampling ratio (max_features): sqrt;

Row sampling ratio (bootstrap): True.

(3) Limiting Gradient Boost (XGBoost): XGBoost is a gradient boost algorithm that performs well in ensemble learning, especially for working with high-dimensional data and complex patterns. In Raman spectral classification, XGBoost improves overall performance by gradually adding weak learners and correcting errors in the previous step. It is efficient, accurate, and interpretable. In this article, the XGBoost parameters were set as follows:

Learning rate: 0.01

Number of trees (n_estimators): 100

Maximum tree depth (max_depth): 10

Column sampling ratio (colsample_bytree): 1

Sample ratio (subsample): 1

(4) Nearest Neighbor Algorithm (KNN): The nearest neighbor algorithm is a simple and intuitive classification algorithm. In Raman spectral classification, KNN classifies based on the distance between samples, that is, the unknown sample is assigned to the class to which its nearest K training samples belong. It is suitable for small data sets, but may be sensitive to noise. In this paper, KNN parameters were set as follows:

Number of n_neighbors: 10;

Distance metric: Euclidean;

(5) Partial least squares discriminant analysis (PLS-DA): PLS-DA is a multivariate statistical analysis method commonly used in Raman spectral classification for reduction and classification tasks. PLS-DA achieves dimensionality reduction by projecting the input variable into a new space and combines it with linear discriminant analysis for classification. It is suitable for feature selection and multicollinearity problems. In this paper, the PLS-DA parameters were set as follows:

Number of latent variables (n_components):10;

(6) Linear discriminant Analysis (LDA): LDA is a classical pattern recognition method that is often used in linearly separable cases in Raman spectral classification. LDA seeks a suitable projection direction to achieve classification by maximizing the intra-class distance and minimizing the inter-class distance. It is suitable for high dimensional data but may have limited effectiveness when dealing with nonlinear problems.
