# Supplementary figures and images for: Rapid identification of lactic acid bacteria at species/subspecies level via ensemble learning of Ramanomes
Source: Front Microbiol. 2024 Apr 8;15:1361180. doi: 10.3389/fmicb.2024.1361180 (PMC11033474; doi:10.3389/fmicb.2024.1361180)

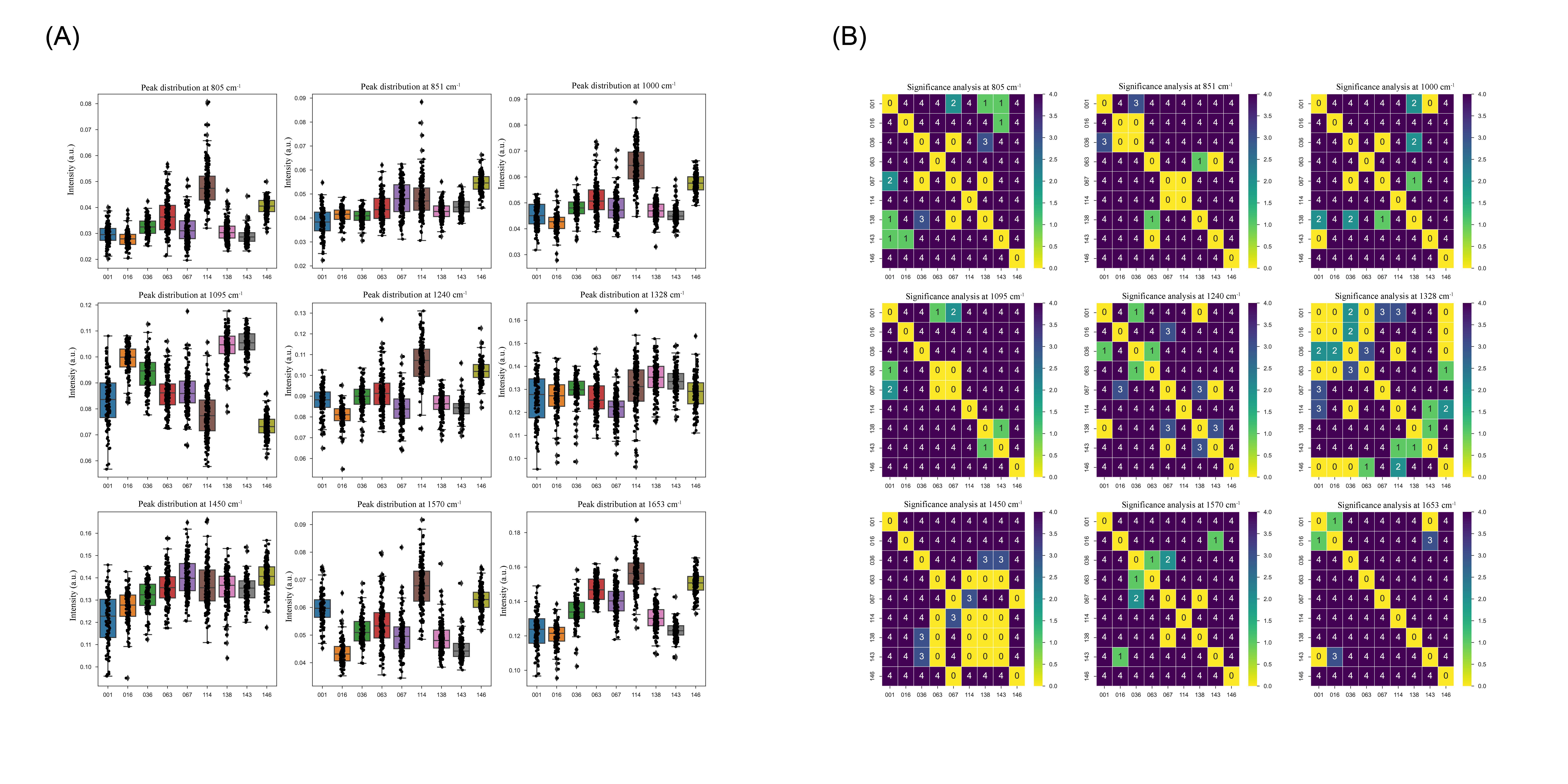

Supplement: Supplementary file 1 [file Data_Sheet_1.zip › Figure S2.JPEG]

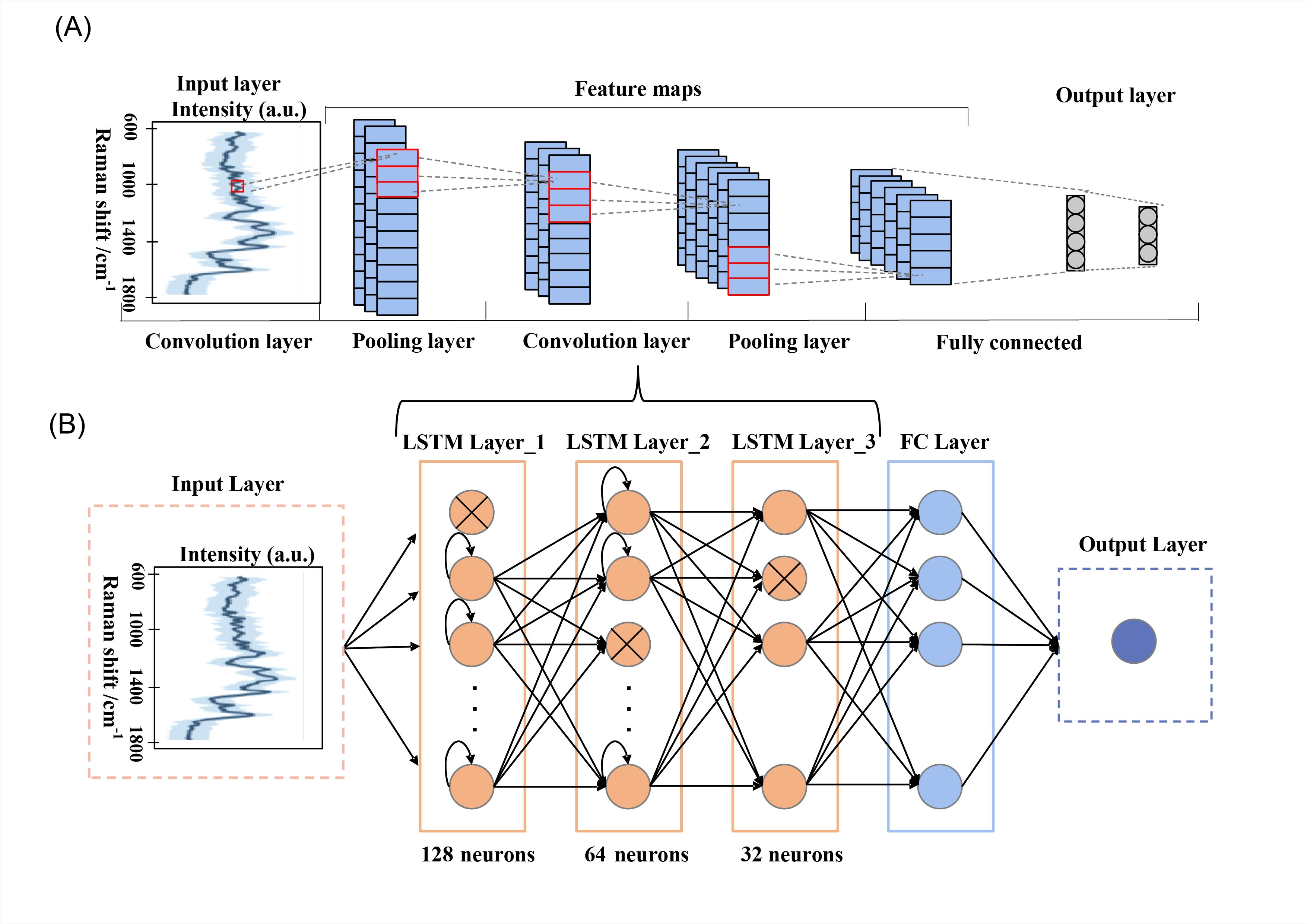

Supplement: Supplementary file 1 [file Data_Sheet_1.zip › Figure S3.JPEG]
